# Social Inequalities in Exposure to Ambient Air Pollution: A Systematic Review in the WHO European Region

**DOI:** 10.3390/ijerph16173127

**Published:** 2019-08-28

**Authors:** Jonathan Fairburn, Steffen Andreas Schüle, Stefanie Dreger, Lisa Karla Hilz, Gabriele Bolte

**Affiliations:** 1 Staffordshire Business School, Staffordshire University, Stoke on Trent ST4 2DF, UK; 2 Department of Social Epidemiology, Institute of Public Health and Nursing Research, University of Bremen, 28359 Bremen, Germany; 3 Health Sciences Bremen, University of Bremen, 28359 Bremen, Germany

**Keywords:** air quality, inequalities, inequities, equity, distribution, environmental justice, environmental inequalities, Europe, deprivation, economic position, preferred reporting items for systematic reviews and meta-analyses (PRISMA)

## Abstract

Ambient air pollution is a long-standing and significant public health issue. The aim of this review is to systematically examine the peer-reviewed evidence on social inequalities and ambient air pollution in the World Health Organization European Region. Articles published between 2010 and 2017 were analyzed in the review. In total 31 articles were included in the review. There is good evidence from ecological studies that higher deprivation indices and low economic position are usually linked with higher levels of pollutants such as particulate matter (particulate matter under 2.5 and 10 microns in diameter, PM_2.5_, PM_10_) and oxides of nitrogen (e.g., NO_2_, and NO_x_). There is also evidence that ethnic minorities experience a mixed exposure in comparison to the majority population being sometimes higher and sometimes lower depending on the ethnic minority under consideration. The studies using data at the individual level in this review are mainly focused on pregnant women or new mothers, in these studies deprivation and ethnicity are more likely to be linked to higher exposures of poor air quality. Therefore, there is evidence in this review that the burden of higher pollutants falls disproportionally on different social groups.

## 1. Introduction

Ambient outdoor air quality is one of the most important environmental factors affecting human health. The World Health Organization (WHO) estimates that 4.2 million deaths occur every year due to ambient air pollution and that 9 out of 10 world’s population live in areas where air pollution exceeds WHO guidelines [1].

The European Environment Agency produced an analysis of air quality in Europe between 2000 to 2016 [2]. This estimated that in 2015 422,000 premature deaths occurred due to PM_2.5_ (particulate matter less than or equal to 2.5 microns in diameter) concentrations (across 41 countries); a further 79,000 were attributed to nitrogen dioxide (NO_2_) levels and 17,700 due to ozone. In Europe, air quality is a major focus of policy for the EU with air quality directives (starting in 1999) implemented by member states at the national level as well as actions at the regional and local level to improve air quality. These have had some success at the macro level with all main pollutants showing total lower levels of emissions in 2016 compared to 2000 with very substantial reductions having been achieved for emissions of sulfur dioxides, carbon monoxide and nitrogen oxides [2].

However, there are long standing concerns about the differential exposure to air pollution between social groups because they contribute to environmental health inequalities. These concerns originated with the growth of the environmental justice movement in the USA in the 1980s and 1990s before being developed further by researchers in Europe in the mid-1990s and early 2000s [3]. An early European study [4] found a very strong social gradient for nitrogen oxides and PM_10_ (particulate matter less than or equal to 10 microns in diameter) in England with the most deprived areas experiencing the worst air quality; including those most likely to be living in areas exceeding the air quality standards set for health. This was a landmark report in the UK leading to development of policy and further research by the Environment Agency and a host of government agencies and charities. For example, the Scottish Government, Forestry Commission Scotland, Scottish Environmental Protection Agency, and Scottish Natural Heritage commissioned a report for Scotland [5] that also found a strong pattern of deprivation and poor air quality for all of Scotland. Other early studies in Europe found similar patterns in Sweden [6], the Netherlands [7], and France [8].

Starting in 2009, a group of international experts coordinated by the WHO has been working on the topic of environmental inequalities. In 2010 a series of papers were published as part of that work [9,10,11,12,13] to inform the preparation and discussion of environmental inequalities at the Fifth Ministerial Conference on Environment and Health in Parma, Italy that year [14]. The Parma Declaration committed governments to a range of actions including Regional Priority 3: To preventing disease through improved outdoor and indoor air quality. A major report which followed that conference was published in 2012 examining environmental health inequalities across the WHO European Region [15].

It has been stated that exposure variation and effect modification are major pathways linking social disparities to environmental health inequalities [10,15]. Socially disadvantaged population groups or areas may experience less favorable environmental living conditions compared to more affluent population groups or areas. Independent from this potential exposure variation, socially disadvantaged groups may have a higher vulnerability leading to more pronounced adverse health effects of a given environmental exposure. Both, exposure differentials and vulnerability differentials, have to be considered in approaches to improve environmental health and to reduce environmentally driven health disparities.

In 2017 the Sixth Ministerial Conference on Environment and Health in Ostrava resolved “to protect and promote the health and well-being of all our people and to prevent premature deaths, diseases and inequalities related to environmental pollution and degradation” [16] (p. 2). Furthermore, one area of action was “improving indoor and outdoor air quality for all, as one of the most important environmental risk factors in the Region, through actions to meet the values of the WHO air quality guidelines in a continuous process of improvement” [16] (p. 3). This paper is one of a series of papers in this topical collection on environmental health equity, which continues the work of the Ostrava Declaration, by reviewing the evidence, and it is also accompanied by an updated assessment report that has analyzed the change over time [17]. The report and several of the associated reviews focus on exposure differentials. This review is especially concerned with the social inequalities in exposure to ambient air pollution.

Many different models and terminology have been used to classify populations according to social characteristics. Examples of commonly used terms include socioeconomic position, socio-economic status, social class and sociodemographic; the definitions of these terms vary both between and within disciplines and from study to study often as a reflection of the data that is available.

In this review, we make use of a modified version of the PROGRESS-Plus framework (Table S1) to classify the studies we have found and refer to the term social dimensions as the overall concept. Social dimensions include all the factors of the PROGRESS-Plus framework e.g., gender, ethnicity, economic position, occupation as well as indices measures that combine some or all of these factors.

The term inequality is used in this review to reflect the measurable and observable differences in exposure to air pollution across the social dimensions without further normative evaluation.

The objective of this systematic review is to synthesise the evidence base on social inequalities in exposure to ambient air pollution in the WHO European Region taking a wide range of social dimensions into account.

## 2. Materials and Methods

This systematic review was registered with the PROSPERO register of systematic reviews (Registration number CRD42018099468) and was carried out using the preferred reporting items for systematic reviews and meta-analyses (PRISMA) statement [18].

Measures on outdoor air pollution include objectively measured air pollutants, proxies (e.g., traffic count data or distance to road), and subjective measures. Objective and subjective measures of noise pollution are not treated as proxies for air pollution (social inequalities in noise pollution are covered in a related systematic review [19]).

### 2.1. Definition of Social Dimension Characteristics

Use was made of a modified version of the PROGRESS-Plus framework (see Table S1) adapted to the research question of this review for identifying a broad range of individual and composite social dimension characteristics that were considered in the review as well as indices. Our main hypothesis is that social dimensions represented by characteristics such as deprived or socially disadvantaged populations or areas have poorer air quality.

In terms of individual social dimension characteristics these were usually but not always reported on in comparison to a reference group in the individual papers. For example, with ethnicity the studies examined whether ethnic minorities had poorer air quality than the white or mainstream population. In the case of the social dimensions age and gender, old people, compared to other age groups, and females, compared to males, were regarded as socially disadvantaged groups.

### 2.2. Search Strategy

Three electronic databases Scopus, Web of Science and MEDLINE (via Pub Med) were searched on 23 April 2018. A wide range of search terms for air quality and social dimensions characteristics were considered along with inequity and inequality or using environmental justice terms using Boolean operators. In PubMed Medical Subject Headings were also used (see Table S2 for search terms).

### 2.3. Eligibility Criteria for Title, Abstract, and Full-Text Screening

All studies included in the review are original observational studies (cohort, cross-sectional, or ecological) with a quantitative approach, written in English, published in peer-reviewed journals and conducted in or across the 53 WHO European countries [20].

This work provides an update to previous work published in 2010 referred to earlier and as such, search results were restricted to articles published between 1 January 2010 and 31 December 2017. Studies were focused on ambient air quality with point source pollution excluded as they form part of another review [21].

To avoid excluding potentially disadvantaged populations, this review was not limited to a particular population but studies had to provide information about social dimensions characteristics of participants or study regions (measured at individual or aggregated level) according to any PROGRESS-Plus factor (Table S1).

Qualitative studies as well as studies with a research focus on animals and their environment were excluded.

### 2.4. Data Collection and Synthesis

**Figure 1:**
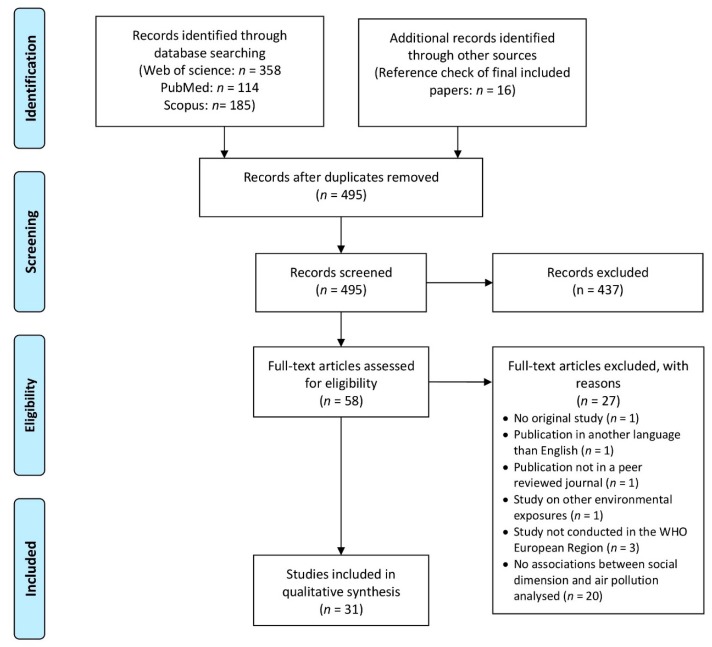
Flow diagram of study selection adapted from the preferred reporting items for systematic reviews and meta-analyses (PRISMA) statement [18].

Two reviewers independently conducted the database search and removing of duplicates separately. The remaining titles and abstracts were screened by two reviewers independently to identify the studies potentially meeting inclusion criteria. We calculated Cohen’s Kappa in order to assess inter-rater reliability agreement for title and abstract screening [22]. Full text of potentially eligible studies was retrieved and systematically assessed for inclusion by two reviewers. Any disagreements between reviewers on the eligibility of a study was referred to a third reviewer. In addition, the reference lists for all articles considered for data extraction were checked in order to find potentially relevant publications missed by the electronic database searches. For illustrating the study selection, PRISMA guidelines were used to produce Figure 1.

Data from all included studies were extracted by one reviewer using a pre-designed extraction data form and crosschecked by a second reviewer. The data extraction table (Table S3) provides a wide range of data including location(s), sample size, study type, different air pollutants, different social dimension measures (both individual and composite indices), data sources, grouping by social dimension characteristics and different types of analyses for measuring environmental inequalities (descriptive, bivariate or multivariate).

Results of studies were only included in the review if numbers could be found in tables. If results were only presented in a narrative way in the text, but specific numbers were not presented, these were not included in our analysis.

In conformity with the other two systematic reviews on environmental inequalities [19,23] we defined in this review a descriptive analysis as a provision of quantitative resource measures across social dimensions in a cross table without performing a statistical test. We compared the highest to the lowest social group in order to assess if environmental inequalities exist or not. We did not define a specific cut-off concerning the magnitude of difference that had to be reached across social groups for being indicated as environmental inequalities because operationalization of social dimensions and air quality may be too heterogeneous. Bivariate analyses included all statistical methods analyzing bivariate associations between social dimensions and air quality measures. Besides descriptive and bivariate results, we also extracted results from multivariate analyses. However, they may be difficult to compare because our research question does not consider a specific definition of additional adjustment or confounding factors. Multivariate models could therefore differ concerning their inclusion of other independent factors, and potential associations between social dimensions and air quality may be concealed due to different modelling approaches.

In both bivariate and multivariate analyses, results with a *p*-value < 0.05 were defined as statistically significant.

To aid readability we have standardized the data in the main extraction table (Table S3) in the following way: a positive relationship is one in which the low social dimensions (e.g., high deprivation, low income, income support, poverty rate) under consideration is linked to higher levels of air pollutants. The same approach has been used with gender, age, and ethnicity where those values of the social dimension under consideration indicating social disadvantage are linked to higher levels of air pollutants and so represented as a positive relationship.

A negative relationship is one in which the low social dimension categories (e.g., most deprived populations) under consideration is linked to lower levels of the air pollutants. Relationships that were equal or not significant are also recorded.

There is an extremely high amount of heterogeneity across the selected studies. For example, in spatial scale (from very small parts of a street, city level, regions, rural–urban, national scale, and across countries), types and number of analysis (descriptive, bivariate, and multivariate), social dimensions characteristics under consideration as well as a mix of study approaches (e.g., ecological studies, cross-sectional studies as well as panel data). Moreover, in accordance with the research question of our review any kind of bivariate analyses of the association of social dimensions and air quality would be meaningful. Any further adjustment such as mutually adjustment for several social indicators would not give the overall association of a certain social dimension characteristic with the exposure. As such, we were not able to apply a standardized quality assessment tool across the studies, which judges multivariate analyses as superior in terms of risk of bias.

## 3. Results

The three databases used identified 657 records. After duplicates were removed and 16 records were additionally included from the reference check, 495 records were considered for title and abstract screening. 58 articles were considered for full text analysis from which 27 were excluded, 31 articles met the criteria (Figure 1). Inter-rater reliability agreement of title and abstract screening was moderate (Cohen’s Kappa value of 0.68).

### 3.1. Description of Studies

Ambient outdoor air quality is objectively measured in the overwhelmingly majority of studies using either air quality monitoring stations data or modelled grids derived from the air quality monitors. In a few cases, measured traffic levels have been used as a proxy for air quality, this is seen as a good proxy as measured traffic levels are often a component of overall modelled air quality. One study [24] makes use of trace elements collected from lichen and constructs an overall pollution score. Only one study in the review [25] uses a subjective measure of traffic load as proxy for air pollution.

There is a wide range of possibilities for classifying the articles in this review. One approach is to classify the articles between observational studies with individual data (10) and observational studies with aggregated data (ecological studies) (20) and there is one article that uses both types of data [26] which was split for reporting purposes to provide 32 studies in total in the tables.

In 10 of the 11 studies using individual data (Table 1) a range of measured or modelled air quality data were used including Benzene, NO_2_, PM_10_, PM_2.5_, ozone, and an air quality index. One study uses a self-reported traffic load at place of residence. All of the studies make use of single measures of social dimension and three studies use an index. Seven countries all from Western Europe are included in the analysis.

Of the 21 ecological studies (Table 2) in the review, 19 use a direct measure of air quality, whilst two studies use the proxy of road traffic levels. Air quality measures include NO_2_, PM_10_, PM_2.5_, carbon monoxide (CO), nitrogen oxides (NO_x_), non-methane volatile organic compounds (NMVOC), ozone, black carbon, as well as various air quality indices. In six of the studies only single measures of social dimensions are used, in nine of the studies only index measures are used and in six studies both types of measure are used. In terms of spatial coverage, 11 studies cover the countries in the UK (Wales, Scotland, England, and Norther Ireland), seven studies come from France, 2 from Spain, 2 from Italy, 1 from The Netherlands. Three of the studies [27,28,29] cover a wider range of countries although using different spatial unit scales. Eleven of the studies make use of bivariate analysis and eight have used multivariate analyses.

In Table 3, Table 4 and Table 5, we synthesize the data using the categories from the modified PROGRESS-Plus framework (Table S1). The term any evidence indicates the presence of any results in the category, the term ‘preponderance of evidence’ is used which refers to the counting of results without any weighting and without differentiation between the single air pollutants.

Within the category economic position, the majority of measures are directly related to material resources (e.g., income measures). We have three that are based on occupational classifications, which in some disciplines these could be described as socio-economic status, however here they are all grouped under economic position (but detail is provided in Table S3 to identify the different variables being used).

### 3.2. Associations between Social Inequality Characteristics and Air Quality

#### 3.2.1. National or International Studies with Complete Population Coverage Using Sub-National Spatial Units

Table 3 provides the detail on seven ecological studies either with a national country coverage or with coverage across different countries. Three of these studies used region level spatial units and the other four used very small spatial units allowing a very detailed distribution to be created for those countries. These types of studies provide a wealth of interesting information and raise some interesting methodological issues.

The first broad finding is that all seven studies support the contention that higher exposure to air pollutants is linked to lower social dimensions. In Wales [39] the most deprived areas are exposed to the highest levels of NO_2_, PM_10_, and PM_2.5_ and a similar finding for all but one type of particulate is found for England using postcode data [45].

In a comparison of The Netherlands and England using small area spatial units very similar results are found in the bivariate analysis for NO_2_ and PM_10_ [41]. In both countries, higher exposure levels are found where the population has higher proportions of income support recipients or non-white populations. Neighborhoods with greater than 20% non-white populations had significantly higher levels of PM_10_ (4.2 μg/m^3^) NO_2_ (13.5 μg/m^3^) in England, and the Netherlands PM_10_ (1.4 μg/m^3^) and NO_2_ (10.4 μg/m^3^).

In the multivariate analysis for England for both pollutants, income support and non-white populations are again found to be significant whereas population over 65 and children are negatively associated with higher levels. In the Netherlands only the non-white population is found to be positively associated with higher exposure levels, income support recipients and children are negatively associated. The study includes a detailed set of results at both the city and regional levels (see Table S3 for more details).

In a study examining all of Great Britain [46], deprivation is consistently linked with negative impacts for exposure to PM_10_ and NO_2_. The study includes the results of change over time between 2001 and 2011 (in this case the air quality data and the social dimension data (indices) are for the same years). Improvements in NO_2_ are negatively related to deprivation, whilst increases in PM_10_ are positively related to deprivation. Exceedances of the air quality limit for PM_10_ and NO_2_ in both 2001 and 2011 are positively linked to higher deprivation. In addition, whilst exposure values have fallen overall over the time period the inequity between most deprived and most affluent has actually increased.

At the regional level in France, links between pollutants and poverty are found [44] whilst in Italy in the highest air pollutant areas exposure is linked to higher proportions of children and female-headed households [43].

In a Europe wide study [28] across 27 countries using regional data and across all years between 2004 to 2008 PM_10_ levels are statistically significantly higher in households with the lowest income. However, the data shows again a U-shaped pattern with the highest level in the lowest income quintile with values lower in quintiles 2, 3, and 4 before generally increasing again at quintile 5. The data also states that the biggest reduction in PM_10_ over time occurred in quintile 1. In a stratification of the data into Western Europe and Eastern Europe, differences emerge. In Western Europe regions Q5 quintiles experience the highest levels of PM_10_ whilst in Eastern Europe the opposite pattern is found.

#### 3.2.2. Ecological Studies at the City or Regional Level

The combined results for several social dimensions of studies at the city or regional level show similar results to the national studies (Table 4). Of the 11 studies with indices data, eight show mainly a positive link with air pollution. For the four studies using economic position two show mainly a positive link with air pollution and the other two mixed results.

The link between social dimension data occurs with economic position in the Asturias in Spain for NO_2_ [42], with deprivation in Dunkerque, France for an air pollution index [24], with deprivation in Lille for NO_2_ [50] and Marseille for NO_2_ [52] although not in Lyon or Paris [50,51]. However, a descriptive result for PM_2.5_ is found in both Grenoble and Lyon for deprivation [47]. In Glasgow [49] deprivation is linked to higher exposure of both NO_2_ and PM_10_.

In Madrid and Barcelona [48] ethnicity is also examined. For Madrid NO_2_ is positively related to areas with higher levels of Asian and Latin Americans ethnic groups but negatively associated with European and African ethnicity (numbers of young children are also negatively associated with NO_2_). In Barcelona, areas with higher levels of four ethnic groups (African, Asian, Latin American, and European immigrants) are positively associated with higher exposure to NO_2_ when compared to the general population. There is also some evidence for Lille [52] for a link between immigrants and NO_2._ exposure.

In the Asturias, Spain [42] a multivariate analysis finds only one minor result linking either NO_2_ or benzene to social dimensions: for benzene only and only in the oldest age group there is a negative association.

#### 3.2.3. Results for Studies Using Individual Level Data

The associations of social dimension characteristics with exposure to air pollutants in studies with individual level data are given in Table 5.

The link between education and exposure for studies using individual data is largely the same as the ecological studies, very mixed tending towards non-significant or neutral.

The patterns for economic position and indices are also similar to the ecological studies although the evidence is generally weaker in studies with individual data. These factors are more likely to be linked to higher exposure for more deprived groups and those with a lower economic position.

In France [37] higher exposure to pollutants (PM_10_, PM_2.5_, NO_2_) is clearly linked to deprivation in nearly all settings and analyses with some small exceptions in rural areas. However, Bertin [30] finds a negative relationship between NO_2_ and neighborhood deprivation in Northern France.

In London [26] individuals with lower grade jobs in the civil service are positively related to higher levels of Nitrogen oxides, but education and household income were not significant.

Analysis of 16 cities in Western Europe [29] which included some data at the individual level found higher educated people are exposed to higher levels of NO_2_ and the same pattern was found for occupational class. However, when the model is adjusted for neighborhood characteristics these factors are not significant and the actual relationship is between neighborhood levels of unemployment, which is positive.

In Valencia [35] a series of analysis reports that younger mothers and mothers from Latin America are exposed to higher levels of NO_2_ both at home and in work.

Vrijheid et al. in working in three cities in Spain find only two statistically significant results amongst pregnant mothers; in Valencia social class is linked to higher exposure for NO_2_ and in Sabadell education levels are inversely linked to higher levels of NO_2_ [38]. Another study in Spain [31] uncovers no relationship between NO_2_ and any of the social dimension variables. There is evidence that the oldest mothers are exposed to lower levels of benzene but this is only significant for the oldest category.

In Southern Sweden [36] a large study of 81,000 births finds a descriptive relationship between higher levels of NO_2_ and non-Nordic mothers, but no relationship with maternal age.

Finally, in a very large study (pop 4.6 million) [33] for Switzerland amongst the population over 30 years of age, Huss et al. report a descriptive pattern between PM_10_ and being unemployed or foreign nationals (the same groups are also found to be living closer to main roads). A negative pattern is found for tertiary education and there is no pattern for age or gender.

## 4. Discussion

The main results of this review are presented in three tables reflecting the spatial extents of the studies and the type of data used: the ecological studies at the national level, ecological studies at the regional level and studies using individual data. This is an important consideration in air quality studies, as results are known to vary between the three study types.

As long ago as 2004 WHO noted “Thresholds are in principle an appealing concept that has also been used in defining air quality policies such as justifying the numerical value of air quality limit values. Nevertheless, recent epidemiological studies investigating large populations have consistently been unable to establish such thresholds, in particular for particulates and ozone. Rather they show effects at the level studied.” [55] (p. 8). Recognition of this fact is what underlines the tightening of air quality standards over time by the EU. Differences in exposure between groups will lead to differences in morbidity and mortality. This is an especially important point to remember when examining the studies in Table 3, which cover the entire population but may only use descriptive analysis.

The findings from both types of ecological studies and studies using individual data show similar patterns for some types of social dimension indicators. Index measures and economic position are fairly consistent in showing that high exposure to poor air quality is linked to deprivation and lower economic position. Fecht et al. provide quantification of some of these differences: in England [41] on average the most deprived had NO_2_ levels that were 7.9 μg/m^3^ higher than compared with the least deprived decile, PM_10_ 2.6 μg/m^3^ higher, in The Netherlands the results were 0.3 μg/m^3^ for PM_10_ and 6.1 μg/m^3^ for NO_2_.

Air quality often exhibits a social gradient, although the most deprived areas have the highest levels of poor air quality, the least deprived areas also experience higher levels of air pollutants than some other social groups. As such, the pattern of air pollution is often described as a U-shaped. This pattern is seen in national studies, which have used small area statistics [4,5]. This is an important consideration to remember when analyzing studies that have been carried out at the city or regional level. The variation in results between cities may in part reflect the status or economic success of that city. Capital cities and successful first order cities often attract more people of a higher economic position who also tend to have better education qualifications as well. Fecht et al. [41] show that virtually all of the population (regardless of composition) in London, Rotterdam, and the Hague were exposed to mean annual pollutants above the then European directive limit for NO_2_. As such the Queen of England experiences some of the worst air quality in the UK when she is in residence in Buckingham Palace (she does of course have several country residences to escape to when pollution is especially poor). However, even the city and regional studies in this study show that areas with higher indices of deprivation and economic position data tend to be associated with higher levels of air pollutants. This maybe because many second order cities especially (post-industrial) in Europe have been stagnating economically and that cities in general almost always have higher levels of air pollutants than rural areas (with the exception of ozone as a pollutant).

In terms of change over time in Britain, Mitchell et al. [46] find that in 2001 the mean concentrations for PM_10_ in the most deprived areas was 10.5% higher than the least deprived areas, however by 2011 this had increased to 14.2% illustrating that in terms of equity the situation had got worse.

A more recent study in nine European Metropolitan areas also supports our general findings. In that study, higher exposure was observed in areas with a higher proportion of people born outside the EU28 or in areas with higher unemployment [56].

Ethnicity in this review as represented by minority groups, immigrants or foreign-born mothers does appear to be linked to higher levels of exposure in general, but even here it varies according to the ethnic group. Other studies have suggested a link between ethnicity and deprivation combining to have a negative impact on some ethnic groups in Leicester for PM_10_ and linking this directly to health outcomes [57,58,59]. Afro-Caribbean and certain South Asian groups were associated with higher levels of PM_10_, Indian ethnicity is negatively associated with PM_10_. Furthermore, in many Western European countries, people with experience of migration from countries of the global south have a greater chance to experience financial hardship and deprived living conditions.

Education is examined in six studies in this review and show a very mixed set of results both within and between studies. In most instances individual educational level is a suitable indicator for material resources [60]. We do not have an explanation for the observed mixed results, educational level might have a different meaning in terms of living conditions across studies and countries.

Place of residence in terms of rural or urban can have an impact on the association of a social dimension with exposure of pollution which can provide a further stratification of the data. For most pollutants, the levels are higher in urban areas as the sources are traffic and industry. However higher ozone levels are usually found in rural areas. Place of residence (Table S5) in studies is usually defined by rural/urban characteristics, the results amongst three of the studies on these characteristics is surprisingly very mixed. In Northern France in both rural and urban areas there is an inverse relationship between deprivation and pollution [30]. In England, the reverse pattern is found for pollutants with the exception of ozone, which is found in less deprived areas [45]. In France, deprived areas in large cities, small city centers and suburban areas have higher levels of pollutants, with the results being mixed for rural areas [37].

It does seem odd that there are so few national ecological studies using small area data to examine the social inequalities of air quality or environmental factors in general for the WHO European Region. The EU for example mandates air quality data to be collected and reported and air quality data is available on a 1 km grid for Europe. The problem seems to be the availability of small area statistics with good socio-economic, socio-demographic or index data to carry out such analyses, but there are a range of other issues that are also preventing this work [61].

This leads directly to the issue of the scale of data units being used in any study. A recent report from the European Environment Agency [62] states “The geographic extent and spatial granularity of the analysis is probably the most important factor affecting both its ability to detect associations between pollution exposure and social indicators, and the interpretation of patterns detected” (p. 58). That study looked at three levels of scale nomenclature of territorial units for statistics (NUTS) 3, NUTS 2, and urban areas, and it provides a useful example of results varying across Europe for larger spatial units.

As already mentioned in the introduction, two mechanisms might contribute separately or in combination to social inequalities in health related to exposure to air pollutants: Social differences in the extent of exposures as was studied in this review on one hand and social differences in vulnerability, that is effect modification by social dimensions and thus increased susceptibility to health risks from air pollution [63].

There have been indications for effect modification by economic position, gender, or age [64,65,66]. Explanations for vulnerability differentials are pre-existing health problems leading to increased susceptibility, other detrimental exposures associated with a lower social position and impairing the health status (e.g., occupational exposures), or socially different possibilities to cope with exposure to air pollutants. For example, Forastiere et al. [67] analyzed short-term effects of PM_10_ on mortality in Rome. Although exposure was higher among those with a high economic position, the effect of PM_10_ on mortality was more pronounced among the residents with a low economic position. The authors explain this discrepancy with a higher proportion of chronic diseases among the residents with low economic position and with the coping possibilities of the residents with a high economic position, who more often had a secondary residence in rural regions with a better air quality.

### 4.1. Limitations

The WHO European Region consists of 53 countries, yet the studies in the review are overwhelmingly from Western Europe, indeed dominated by studies from the UK, France, Spain, and Italy. These results reflect not just the place of peer review publication, but also the issues of data collection, availability and access across countries as well as political interest and support for research in the subject area.

A further limitation of this review is that of language, as all studies were in English in peer review journals—the dominance of English peer review papers have been discussed [68] with specific reference to public health studies.

A series of systematic reviews in different languages would almost certainly provide more data for some countries (e.g., France, Spain, Italy, and Germany) although we already have some studies for these countries in English. Ecological studies for air quality exposure by indices or other social dimensions are certainly possible for most if not all of the EU countries as monitoring of air quality has been required for some time under Air Quality Directives.

For ecological studies, the size of the spatial unit can have an impact on the overall results. As the size of spatial unit increases the variation in the data decreases—this happens as data points are combined to produce averages for the spatial units under consideration. This can explain why some social dimensions produce statistically significant results in some studies but not in others. This is a long standing and well-known issue with ecological studies which is known as the Modifiable Areal Unit Problem.

One common feature to studies in this review is that they are often reliant on disparate datasets, which have been collected for different purposes and over different times. This area of work has been characterized by the use of Geographic Information Systems, which allow the integration of data from different domains such as environment, health, social, or economic. However, the fact remains that in many of the studies different data has been created or collected in different years and then integrated together meaning there is an assumption that populations are both static and that any movement of people out of an area is being replaced by similar people moving into an area.

The issue of availability of data can also be seen in regard to the dates of publication for the studies. In many of the studies the date of publication is at least a decade (and often more) after the data was collected. As such, we are often looking at a historical pattern of environmental exposure and the social dimensions under investigation.

The magnitude of inequalities were not analyzed due to the heterogeneity of study populations, social dimensions and different exposure assessments [69,70]. For bivariate analysis we have used the standard 0.05 cut-off point but there has been increasing concerning in the sciences about the use of these measures [71,72].

Finally, we did not apply a standard quality assessment of the included studies. This is not just due to the heterogeneity of the studies included in this review, but also due to our research question. Any quality assessment tool rating multivariate analyses higher in terms of risk of bias due to confounding would not take into consideration that this review focuses on the overall association of a social dimension with air pollution levels.

### 4.2. Strengths

The study has followed recognized standards and protocols as far as possible: pre-registered on PROSPERO, a rigorous selection process using PRISMA and use of a recognized framework PROGRESS-Plus. This is a comprehensive review using an equity lens on the social dimensions of air pollution. We have included all types of studies as well as a wide range of air pollutants and social dimensions. Thus, a considerable number of studies could be analyzed in this review.

The level of heterogeneity whilst problematic in terms of potential grading of quality does illustrate the wide variety of methods that can be used across a range of spatial scales to investigate the social distribution of air quality which is important for different policy makers and researchers.

### 4.3. Implications for Practice

An extensive literature exists for tackling air pollutants, a recent review provides some case studies [73]. What is clear from this review is that even though air quality has improved for most people using universal or population wide mechanisms (e.g., improvements in car standards, movement to cleaner fuel sources for mass energy production) targeted actions will be needed to address the current inequities that deprived areas and socially disadvantaged population groups, respectively, face especially in cities.

There is still the need for systematic evaluation of equity impacts of interventions to reduce air pollutant exposure [74,75] to assess whether any measurements to mitigate air pollutants would decrease or even increase the social gap in exposure.

### 4.4. Implications for Further Research

Most of the studies in this review are cross-sectional and in some cases out of date (using data from the early 2000s and we know there has been an overall improvement for many air pollutants in general over the last decade). Regular and systematic monitoring is needed to account for temporal change in exposure amongst the population.

Many of the studies are only carried out at a city or regional level. The lack of nationwide studies is a particular concern. It should be entirely possible for countries to produce regularly updated ecological studies covering the whole country on a small area spatial unit. Such an approach would always allow a further aggregation of spatial units to a large spatial scale if it were required. It should not be left to interested academics to produce such studies but should be seen as a regular activity by governments and their agencies as a means of monitoring their commitments made under the Ostrava declaration. Environmental epidemiological studies should further integrate personal air pollutant exposure monitoring, contextual and individual data on social dimensions, and collect data on daily routines in space and time of the study participants to be able to analyze social inequalities in air pollutant exposure more reliably.

## 5. Conclusions

Social inequalities in exposure to ambient air pollution are evident across Western Europe, there is very little data available for Eastern Europe in this review. Indices of deprivation generally show that for most pollutants the most deprived areas experience the worst air quality. This pattern is replicated with economic position data as well. Other social dimensions also suggest cause for concern with ethnicity and other vulnerable groups at risk of higher levels of air pollutants. There is a lack of regular, countrywide studies in most of the WHO European Region examining the issues of environmental equity between social groups. Regular and consistent monitoring of air quality by social dimensions would be one way for governments to demonstrate the commitments they made in the Ostrava Declaration of 2017. This would help to improve and target policy for the health of their populations.

## Figures and Tables

**Table 1:** Description of 11 studies using individual data. (particulate matter under 2.5 and 10 microns in diameter, PM_2.5_ and PM_10_, respectively).

Author, Year	Air Quality		Social Dimension Measures		Type of Analysis			
	Objective	Subjective	Single Measures	Index	Descriptive	Bivariate	Multivariate	Country
Bertin et al. 2015 [30]	NO_2_		X	X	X			France
Fernandez-Somoano and Tardon 2014 [31]	NO_2_, Benzene		X		X	X	X	Spain
Ferrero et al. 2017 [32]	Benzene		X				X	Spain
Goodman et al. 2011b [26]	NO_x_		X	X		X	X	England
Huss et al. 2010 [33]	PM_10_		X		X			Switzerland
Lejune et al. 2016 [34]	Air Quality index		X			X		Belgium
Llop et al. 2011 [35]	NO_2_		X		X		X	Spain
Malmqvist et al. 2011 [36]	NO_x_		X		X			Sweden
Ouidir et al. 2017 [37]	PM_2.5_, PM_10_, NO_2_		X	X		X	X	France
Scharte and Bolte 2013 [25]		Self-reported traffic load at place of residence	X			X		Germany
Vrijheid et al. 2012 [38]	NO_2_		X				X	Spain

**Table 2:** Description of 21 ecological studies. NMVOC: non-methane volatile organic compounds.

Author, Year	Air Quality		Social Dimension Measures		Type of Analysis			
	Objective		Single Measures	Index	Descriptive	Bivariate	Multivariate	Country
Brunt et al. 2016 [39]	NO_2_, PM_10_, PM_2.5_	PM_2.5_		X	X			Wales
Castellano et al. 2010 [27]	CO, NO_x_, NMVOC		X	X			X	OECD * countries
Cesaroni et al. 2010 [40]	Road traffic levels		X	X	X		X	Italy
Fecht et al. 2015 [41]	PM_10_, NO_2_		X	X		X	X	England, The Netherlands
Fernandez-Somoano et al. 2013 [42]	NO_2_		X	X	X		X	Spain
Germani et al. 2014 [43]	AQ index		X			X	X	Italy
Goodman et al. 2011a [26]	NO_x_		X	X		X	X	England
Lavaine 2014 [44]	NO_2_, PM_10_, ozone		X		X			France
Milojevic et al. 2017 [45]	Particulates (range), ozone			X	X			England
Mitchell et al. 2015 [46]	NO_2_, PM_10_			X	X			Great Britain
Morelli et al. 2016 [47]	PM_2.5_			X	X			France
Moreno-Jimenez et al. 2016 [48]	NO_2_		X		X	X		Spain
Morrison et al. 2014 [49]	NO_2_, PM_10_			X		X		Scotland
Occelli et al. 2016 [24]	AQ Index			X	X	X		France
Padilla et al. 2013 [50]	NO_2_			X	X	X		France
Padilla et al. 2016 [51]	Proximity to high traffic roads			X		X		France
Padilla et al. 2014 [52]	NO_2_		X	X	X		X	France
Richardson et al. 2013 [28]	PM_10_		X		X	X		East and West Europe
Rivas et al. 2017 [53]	PM_10_, PM_2.5_, Black carbon, Ultrafines			X	X	X		England
Teman et al. 2017 [29]	NO_2_		X				X	7 European countries
Xie and Hou 2010 [54]	AQ Index		X		X			England

* OECD—Organisation for Economic Co-operation and Development.

**Table 3:** National or international studies with sub-national data units.

Social Dimension	Any Evidence			Preponderance of Evidence			
	⊕	⊖	= or n.s.	⊕	⊖	Mixed	= or n.s.
Ethnicity	[41,43]	[43]		[41]		[43]	
Occupation	[44]			[44]			
Gender	[43]			[43]			
Education		[43]			[43]		
Economic position	[39,41,44]	[41,43,44]		[39,41,44]	[43]		
Indices	[28,45,46]	[45]	[45]	[28,45,46]			
Age	[41,43]	[41,43]	[41]			[41,43]	
Other vulnerable groups							

Note—No studies for the categories Religion, Social Capital, Disability, Sexual Orientation. For place of residence as further stratification variable see Table S4. “=” = no social unequal distribution of air pollution or n.s. = not significant. “⊕” = lower social dimension groups (e.g., more deprived populations) have higher air pollution levels. “⊖” = lower social dimension groups (e.g., more deprived populations) have lower air pollution levels.

**Table 4:** Ecological studies at the city or regional scale.

Social Dimension	Any Evidence			Preponderance of Evidence			
	⊕	⊖	= or n.s.	⊕	⊖	Mixed	= or n.s.
Ethnicity	[48,52]	[48,52]	[52]	[48]		[52]	
Occupation	[29,52]	[29,52]	[29,52]			[29]	[52]
Gender			[27]				[27]
Education	[26,29,54]	[26,29,40,42,54]	[26,29,40,42,52]		[40]	[26,29,54]	[42,52]
Economic position	[26,52,54]	[52,54]	[52]	[26]		[52,54]	
Indices	[24,26,27,29,42,47,49,50,51,52,53]	[27,29,40,52]	[27,29,40,53]	[24,26,27,42,47,49,50,51,52]	[40]	[29]	[53]
Age	[40,48]	[48]	[27,48]	[40]		[48]	[27]
Other vulnerable groups	[52]	[52]	[52]			[52]	

Note—No studies for the categories Religion, Social Capital, Disability, Sexual Orientation. For place of residence as further stratification variable, see Table S4. “=” = no social unequal distribution of air pollution or n.s. = not significant. “⊕” = lower social dimension groups (e.g., more deprived populations) have higher air pollution levels. “⊖” = lower social dimension groups (e.g., more deprived populations) have lower air pollution levels.

**Table 5:** Studies using individual level data.

Social Dimension	Any Evidence			Preponderance of Evidence			
	⊕	⊖	= or n.s.	⊕	⊖	Mixed	= or n.s.
Ethnicity	[32,33,35]		[31,38]	[32,33,35]			[31,38]
Occupation	[26,33]		[31]	[26,33]			[31]
Gender			[33]				[33]
Education	[37]	[26,33,37,38]	[26,31,35,37,38]		[33]	[26,37]	[31,35,38]
Economic position	[26,31,34,35,38]		[26,31,35,38]	[26,34]			[31,35,38]
Indices	[26,36,37]	[30,36,37]	[36]	[26,36,37]	[30]		
Age			[33]				[33]
Other vulnerable groups	[25,35]		[31,35,37]	[25,35]			[31,37]

Note—No studies for the categories Religion, Social Capital, Disability, Sexual Orientation. See Table S4 for place of residence as further stratification variable. “=” = no social unequal distribution of air pollution or n.s. = not significant. “⊕” = lower social dimension groups (e.g., more deprived populations) have higher air pollution levels. “⊖” = lower social dimension groups (e.g., more deprived populations) have lower air pollution levels.

## Supplementary Materials

The following are available online at https://www.mdpi.com/1660-4601/16/17/3127/s1, Table S1: Modified PROGRESS-Plus framework, Table S2: Search terms and medical subject headings in PubMed, Table S3: Data extraction table for the studies, Table S4: Stratification by place of residence, Table S5: PRISMA checklist.
