# Supplementary material for: Social Inequalities in Exposure to Ambient Air Pollution: A Systematic Review in the WHO European Region
Source: Int J Environ Res Public Health. 2019 Aug 28;16(17):3127. doi: 10.3390/ijerph16173127 (PMC6747075; doi:10.3390/ijerph16173127)
Supplement: Supplementary file 1 [file ijerph-16-03127-s001.pdf]

Table S1 Modified PROGESS-Plus Framework

Socioeconomic/sociodemographic characteristics considered in the systematic review using PROGRESS-Plus as conceptual framework (see reference below). These social factors may contribute to unequal health status within and between populations.

The PROGRESS-Plus Framework has been modified according to the research question of this review.

PROGRESS	
Ethnicity	Ethnic background
Occupation	Professional, skilled, unskilled, unemployed, etc
Gender	Male or female
Religion	Religious background
Education	Years in and/or level of education attained
Social capital	Neighbourhood/community/family support
Economic position	Income, means tested benefits/welfare, affluence measures, etc
PLUS	
Indices	Index of deprivation at a regional level using aggregated data (e.g. income-related, education, occupation, elements of place residence) or index at individual level using (e.g. education, income, occupational status of individuals)
Age	Age range or certain age groups
Disability	Existence of physical or emotional/mental disability
Sexual orientation	Heterosexual, gay, lesbian, bisexual, transgender
Other vulnerable groups	School non-attenders, young person in criminal justice system, victims of abuse, teenage parents

The term 'economic position' is used in this table to encompass the key elements of what in UK research has traditionally been termed 'social class'.

[Kavanagh, Equity Update 2008 (Cochrane Health Equity Field and Campbell Equity Methods Group)]

Further reference:

O'Neill J, Tabish H, Welch V, Petticrew M, Pottie K, Clarke M, et al. Applying an equity lens to interventions: using PROGRESS ensures consideration of socially stratifying factors to illuminate inequities in health. J Clin Epidemiol. 2014;67(1):56–64

Supplementary Table 2 – Search terms and medical subject headings in PubMED

PubMed Advanced Search

Air pollution

("sociological factors"[MeSH Terms] OR disadvantaged[All Fields] OR disadvantage[All Fields] OR deprived[All Fields] OR social[All Fields] OR socio*[All Fields] OR sociological[All Fields] OR "vulnerable populations"[MeSH Terms] OR vulnerable[All Fields] OR vulnerability[ALL Fields] OR "psychosocial deprivation"[MeSH Terms] OR psychosocial[All Fields] OR psycho-social[All Fields] OR "socioeconomic factors"[MeSH Terms] OR socioeconomic[ALL Fields] OR socio-economic[ALL Fields] OR deprivation[All Fields] OR sociodemographic[All Fields] OR socio-demographic[All Fields])

AND

("air quality"[Title/Abstract] OR "nitrogen oxides"[Title/Abstract] OR "nitrogen oxide"[Title/Abstract] OR "sulfur dioxides"[Title/Abstract] OR "sulfur dioxide"[Title/Abstract] OR "sulphur dioxides"[Title/Abstract] OR "sulphur dioxide"[Title/Abstract] OR SO2[Title/Abstract] OR "air pollution"[Title/Abstract] OR "air pollutants"[Title/Abstract] OR "air pollutant"[Title/Abstract] OR "particulate matter"[Title/Abstract] OR NO2[Title/Abstract] OR NOX[Title/Abstract] OR PM10[Title/Abstract] OR PM2.5[Title/Abstract] OR ozone[Title/Abstract] OR O3[Title/Abstract] OR soot[Title/Abstract]) OR "ultrafine particles"[Title/Abstract])

AND

(inequality[Title/Abstract] OR inequity[Title/Abstract] OR inequities[Title/Abstract] OR inequalities[Title/Abstract] OR unequal[Title/Abstract] OR "environmental justice"[Title/Abstract] OR "environmental injustice"[Title/Abstract])

AND

("2010/01/01"[Date - Publication] : "2017/12/31"[Date - Publication])

Web Of Science

Air pollution

TS=(disadvantaged OR disadvantage OR deprived OR social OR socio* OR sociological OR vulnerable OR vulnerability OR psychosocial OR psycho-social OR socioeconomic OR socio-economic OR deprivation OR sociodemographic OR socio-demographic)

AND

TS=(“air quality” OR “nitrogen oxides” OR “nitrogen oxide” OR “sulfur dioxides” OR “sulfur dioxide” OR “sulphur dioxides” OR “sulphur dioxide” OR SO2 OR “air pollution” OR “air pollutants” OR “air pollutant” OR “particulate matter” OR NO2 OR NOX OR PM10 OR PM2.5 OR ozone OR O3 OR soot OR “ultrafine particles”)

AND

TS=(inequality OR inequity OR inequities OR inequalities OR unequal OR “environmental justice” OR “environmental injustice”)

AND

PY=(2010-2017)

Restrict results by language and document types (to be selected manually)

- English
- Article

Scopus – Advanced search

Air Pollution

ALL(disadvantaged OR disadvantage OR deprived OR social OR socio* OR sociological OR vulnerable OR vulnerability OR psychosocial OR psycho-social OR socioeconomic OR socio-economic OR deprivation OR sociodemographic OR socio-demographic) AND TITLE-ABS-KEY(“air quality” OR “nitrogen oxides” OR “nitrogen oxide” OR “sulfur dioxides” OR “sulfur dioxide” OR “sulphur dioxides” OR “sulphur dioxide” OR SO2 OR “air pollution” OR “air pollutants” OR “air pollutant” OR “particulate matter” OR NO2 OR NOX OR PM10 OR PM2.5 OR ozone OR O3 OR soot OR “ultrafine particles”) AND TITLE-ABS-KEY(inequality OR inequity OR inequities OR inequalities OR unequal OR "environmental justice" OR "environmental injustice") AND LANGUAGE(english) AND PUBYEAR > 2009 AND PUBYEAR < 2018 AND DOCTYPE(ar) AND NOT INDEX (medline)

Supplementary Table S3 – Main data extraction table

**Authors**	***Place of study***	***Unit of analysis***<br><br>***(Study population and sample size)***	**Study type**	***Measurement of exposure / operationalisation of environmental exposure***	***Method for environmental exposure measurement***	***Social dimensions***	***Data sources social dimensions***	***Quantification of social inequalities***	***Results on environmental inequalities in symbols***
Bertin<br>2015	France<br><br>Brittany	Indiviudal<br>2,509 pregnant women	Study with individual data, Panel data study 2002 – 2006 (PELAGIE mother-child cohort)	Objective<br><br>No2 – 100 metre grid matched with individual home address	GIS	Indices<br>(Neighbourhood deprivation)	Questionnaire<br><br>Neighbourhood deprivation index	Absolute	**Description**<br><br><u>NO2</u><br>⊖ neighbourhood deprivation (rural and urban) (n.l. in middle groups)
Brunt et al 2016	Wales	Aggregated level:<br>1,909 Lower Super Output Areas for all of Wales average pop 1,600	Ecological study, cross- sectional<br><br>Note the entire population is in the study	Objective:<br><br>No2, PM10, PM2.5 on a 1km grid 2011-2013 averaged	GIS	Economic position<br>(Income)	Income- Deprivation domain data from the Welsh Index of Multiple Deprivation<br>LSOAs allocated to quintiles -	Absolute and relative	**Description**<br><br><u>NO2, PM10, PM2.5</u><br><br>⊕ Income deprivation (n.l. in middle groups).<br><br>U shaped NO2 and PM10, PM2.5
Castellano et al 2010	OECD countries	Country level comparison s	Ecological study,<br><br>Panel study 1995-2005	CO, NOx, NMVOC, 1995-2005 OECD data	Regression model	Gender<br><br>Age<br><br>Indices	World Development Indicators (World Bank)	Absolute	**Multivariate**<br><br>**<u>CO</u>**<br><br>⊕ GDP, GINI<br>ns sex (number of women in legislative bodies, age (population 014)<br><br><u>N0x</u><br><br>⊕ GDP<br>n.s. sex (women in legislative bodies)<br><br>⊖ GINI<br><br>n.s. age (population 0-14)<br><br><u>NMVOC</u><br>⊕GDP<br>ns GINI, sex (women in legislative bodies), age (population 0-14)
Fecht et al 2015	England, Netherlan ds,	Aggregated<br><br>National, region and city level<br><br>England 49,138,831<br><br>Netherland s<br>16,097,060	Ecological study, cross-sectional, using the total population	PM10, NO2, on 100 metre grids modelled in 2001	GIS	**England –**<br>Economic position<br>(Income support recipients)<br>Ethnicity<br>Age<br>Other vulnerable groups (Children)<br>Place of residence<br>**Netherlands**<br>Economic position (Income)<br>Ethnicity (Non Western immigrants)<br>Age	**England –** Income domain from the 2004 Index of Multiple Deprivation, Census<br><br>**Netherlands**<br>Central Bureau of Statistics	Absolute and relative	**Bivariate - correlation co-efficient**<br><br><u>PM10 and NO2 (National level)</u><br><br>⊕ Income support recipients (England and Netherlands, non-white (England and Netherlands), Pop over 65 Netherlands<br><br>⊖ (Netherlands), Pop over 65 (England)<br><br>n.s. children (England)<br><br>**Bivariate mean air pollution**<br><br><u>PM10 (National level)</u>

									⊕ Income support recipients (England and Netherlands), non-white (England and Netherlands), Pop over 65 Netherlands<br><br>⊖ (Netherlands), Pop over 65 (England)<br><br><u>NO2 (National Level)</u><br><br>⊕ Income support recipients (England and Netherlands), non-white (England), children (England) Pop over 65 (Netherlands)<br><br>⊖ Pop over 65 (England), children (Netherlands)<br><br><u>PM10 City level</u><br><br>⊕Income support recipients (Birmingham, Leeds, Liverpool, London, Sheffield, The Hague), non-white (Birmingham, Bristol, London, Sheffield), children (Birmingham, Sheffield)<br><br>⊖ non-white (Leeds), children (Bristol, London, Amsterdam), Pop over 65 (all cities considered)<br><br>n.s. Income support recipients (Bristol, Amsterdam, Rotterdam), non-white (Liverpool), children (Leeds, Liverpool, Rotterdam, The Hague)<br><br><u>NO2 City level</u><br><br>⊕ Income support recipients (Birmingham, Leeds, Liverpool, London, Sheffield, Amsterdam, Rotterdam , The Hague,)<br>Non-white (Birmingham, Bristol, Leeds, Liverpool, London, Sheffield,<br>Children (Bristol, Liverpool, London,<br><br>⊖ Children (Birmingham, Sheffield,<br><br>n.s. Income support recipients (Bristol), Non-white (Amsterdam, Rotterdam, The Hague) , Children (Leeds, Amsterdam, Rotterdam, The Hague)<br><br>**Multivariate**<br><br><u>PM10 (National level</u>)<br><br>⊕ Income support recipients (England), non-white (England, Netherlands)<br>⊖ Income support recipients (Netherlands), children (England, Netherlands), Pop over 65 (England)<br>n.s. Pop over 65 (Netherlands)<br><br><u>NO2 National level</u><br><br>⊕ Income support recipients (England), non-white (England, Netherlands)<br>⊖ Income support recipients (Netherlands), children (England, Netherlands), Pop over 65 (England)<br>n.s. Pop over 65 (Netherlands)<br><br><u>PM10 Regional level</u><br><br>⊕ Income support recipients (East of England, East Midlands, North East, North West, South East, South West, West Midlands, Yorkshire

									Northern NL), Ethnicity (East Midlands, North East, North West, South East, South West, West Midlands, Yorkshire, Western NL, Southern NL) , Children (Southern NL)<br><br>⊖ Income support recipients (Eastern NL, Western NL, Southern NL), Ethnicity (East of England), Children (East of England, East Midlands, North East, North West, South East, South West, Yorkshire, Northern NL, Eastern NL, Western NL) , Pop over 65 (East of England, East Midlands North East, North West, South East, South West, Yorkshire, Northern NL, Eastern NL,<br><br>n.s. Ethnicity (Northern NL, Eastern NL) , Children (West Midlands), Pop over 65 (West Midlands , Western NL, Southern NL)<br><br><u>NO2 Regional level</u><br><br>⊕ Income support recipients (East of England, East Midlands, North East, North West, South East, South West, West Midlands, Yorkshire, Western NL, Southern NL) Ethnicity (East Midlands, North East, North West, South East, South West, West Midlands, Yorkshire, Western NL, Southern NL)<br><br>⊖ Children (East of England, East Midlands, North East, North West, South East, South West, West Midlands, Yorkshire, Northern NL, Eastern NL, Western NL, Southern NL), Pop over 65 (East of England, East Midlands, North East, North West, South East, South West, West Midlands, Yorkshire, Northern NL, Eastern NL, Western NL, Southern NL)<br><br>n.s. Income support recipients (Northern NL, Eastern NL,) , Ethnicity (East of England, Northern NL, Eastern NL,<br><br><u>PM10 (City level)</u><br><br>⊕ Income support recipients (Birmingham, Bristol, Leeds, Liverpool, London, Sheffield, Rotterdam, The Hague), Ethnicity (Birmingham, Bristol, London, Sheffield, Rotterdam, The Hague)<br><br>⊖ Children (Bristol, Leeds, London, Amsterdam, Rotterdam,) , Pop over 65 (Bristol, Leeds, Liverpool, London, Sheffield, Amsterdam, Rotterdam)<br>n.s. Income support recipients (Amsterdam) , Ethnicity (Leeds, Liverpool, Amsterdam), Children (Birmingham, Liverpool, Sheffield, The Hague), Pop over 65 (Birmingham, The Hague<br><br><u>NO2 (City level)</u><br><br>⊕Income support recipients (Birmingham, Bristol, Leeds, Liverpool, London, Sheffield, Amsterdam) , Ethnicity (Birmingham, Bristol, Leeds, London, Sheffield, The Hague)<br><br>⊖ Children (Birmingham, Bristol, Leeds, Liverpool, London, Sheffield,) , Pop over 65 (Birmingham, Bristol, Leeds, Liverpool, London, Sheffield)<br><br>n.s. Income support recipients (Rotterdam, The Hague), Ethnicity (Liverpool, Amsterdam, Rotterdam), Children (Amsterdam, Rotterdam, The Hague) , Pop over 65 (Amsterdam, Rotterdam, The Hague)
Fernandez-Somoano et al 2013	Asturias, Spain	Aggregated level	Ecological study, cross-sectional	Objective<br><br>NO2 50m x 50m grid	GIS	Indices (socio-economic status index based on occupation and activity)	Standard classifications of occupations in Spain	Absolute	**Descriptive**<br><br><u>NO2 (Urban<50%)</u>

		Census tracts (pop approx. 1000-2000)<br><br>Population total – 154,918				Education<br><br>Place of residence<br>[Rural urban (two categories: <50% urban land and >50% urban land)]	Standard classification of education levels in Spain<br><br>Classification of census tracts to urban and rural		⊕ Socio-economic index<br>⊕ Education<br><br>NO2 (Urban >50%)<br>⊖ Socio-economic index<br>⊖ Education<br><br>NO2 All<br>⊕ Socio-economic index<br>⊖ Education<br><br>Bivariate (spatial lag)<br><br>NO2 (Urban<50%)<br>⊕ Socio-economic index<br>n.s. Education<br><br>NO2 (Urban >50%)<br>NS Socio-economic index<br>⊖ Education<br><br>NO2 All<br>⊕ Socio-economic index<br>n.s. Education<br><br>**Multivariate (spatial lag, SE Index and Education)**<br><br>NO2 (Urban<50%)<br>⊕ Socio-economic index<br>n.s. Education<br><br>NO2 (Urban >50%)<br>n.s. Socio-economic index<br>Education<br><br>NO2 All<br>⊕ Socio-economic index<br>n.s. Education
Ferrero A et al 2017	Valencia, Spain	Individuals<br><br>1 year olds – n= 352 and their parents	Study with individual data, Panel study 2006-2007<br>(INMA cohort)	Objective<br><br>Benzene	Benzene samplers inside and outside dwellings over a 15 day period<br><br>Questionnaire on respiratory symptoms over the last 12 months	Ethnicity (Maternal country of birth)	Location data<br><br>Survey data (parental questionnaire)	Absolute	**Multivariate**<br><br>Benzene<br><br>⊕ Children with non-Spanish mothers
Germani et al 2014	Italy	Aggregated<br><br>Provinces n= 103<br><br>Total Italian population not provided.	Ecological study, cross- sectional	Objective<br><br>Different levels of air pollutants (12) which together are combined to create an Air Quality Index using Italian threshold values<br><br>CO2	Emission data with reference to Italian limit values for ambient pollution	Education (percentage of the population which has an undergraduate university degree)<br><br>Economic position<br>(Income data – real household disposable income per capita for each province)<br><br>Gender<br>(Females: Percentage of family households with a female as head of the household)	Census data<br><br>Italian Chambers of Commerce data	Relative	**Descriptive**<br>Air pollution index<br>⊖ per capita income. % with a university degree. % Asian. %Elders<br>⊕ % African. %Children. %female headed households<br><br><br>CO2<br>⊖ Income. University degree. Asian. Pop over 65<br><br>⊕ African. Children. Female headed households<br><br>**Multivariate (marginal effects from ordered probit regression)**<br><br>Air pollution index

						Age<br>(Age 15 to 34<br>Age 35 to 49<br>Age 65+)<br><br>Ethnicity<br>(Percentage of African residents: Percentage of Asian residents)			Low –<br>⊕ university degree,<br>⊖ children, female headed households<br>n.s. Asian, African, elders,<br><br>Medium low –<br>⊖children, female headed households<br>n.s. University degree, Asian, African, elders,<br><br>Medium-high<br>⊕ children, female headed households<br>n.s. University degree, Asian, African, elders,<br><br>High<br>⊕ children, female headed households<br>n.s. University degrees, Asian, African, elders<br><br><u>CO2</u><br><br>Low emission<br>⊖children<br>female headed households<br>n.s. University degrees, Asian, African, elders,<br><br>Medium-low<br>⊖children<br>female headed households<br>n.s. University degrees, Asian, African, elders,<br><br>Medium high<br>⊕children<br>n.s. University degrees, Asian, African, elders, female headed households<br><br>High<br><br>⊕children<br>female headed households<br>n.s. University degrees, Asian, African, elders,
Goodman et al 2011 (a)	London (UK)	Unit postcodes – 186,424<br><br>5,344 Super output areas (pop ca 1,500 per SOA)<br><br>3,654 Individual data	Ecological study, cross-sectional study	NOx on a 20metre by 20 metre grid	Modelled data and GIS	Indices (multiple measures)<br><br>Economic position (multiple measures)<br><br>Education (multiple measures)	Index of Multiple Deprivation<br><br>Individual economic position from the Whitehall II cohort<br><br>Acorn groups (representing income) in some of the analysis	Relative	**Bivariate**<br><u>NOx</u><br><br><u>All of London</u><br><br>⊕ Household income<br>n.s. Education<br><br>⊕ Employment, Education child,<br><br>⊖ Education adult<br><br>**Bivariate with spatial controls**<br><br><u>NOx</u><br>⊕ Overall IMD, IMD (minus outdoor env)<br>IMD subdomains Income, Employment, Education child.<br>⊕ Income (ACORN)<br>⊖ Education Adult<br><br><u>Urban zone results</u><br><br><u>NOx</u><br><br><u>Central London</u>

									⊖ Full IMD score, IMD minus outdoor env, Income domain, Employment domain, Education adult<br>n.s. Education child,<br><br><u>Non-central inner London</u><br>⊕Full IMD score, IMD minus outdoor env, Income domain, Employment domain; Education child,<br><br>⊖ Education adult<br><br><u>Outer London</u><br>⊕ Full IMD score, IMD minus outdoor env, Income domain, Employment domain, Education child<br><br>⊖ Education adult<br><br><u>Outside London</u><br><br>⊕ Education child, Education adult<br><br>n.s. Full IMD score, IMD minus outdoor env, Income domain, Employment domain<br><br>**Multivariate with IMD and Acorn groups**<br><br><u>NOx</u><br><br><u>All of London</u><br><br>⊕ Employment domain,<br>⊖ Education adult<br>n.s. Education child<br><br><u>Central London</u><br>n.s. Employment, Education child, Education adult,<br><br><u>Non-central inner London</u><br>⊕ Employment domain,<br>⊖ Education adult<br>n.s. Education child, ⊖ Education adult<br><br><u>Outer London</u><br>⊕ Employment domain,<br>n.s. Education child,<br>⊖ Education adult<br><br><u>Outside London</u><br><br>n.s. Employment, Education child, Education adult
Goodman et al 2011 (b)	London (UK)	Unit postcodes – 186,424<br><br>5,344 Super output areas (pop ca 1,500 per SOA)<br><br>3,654 Individual data	Study with individual data, cross-sectional study	NOx on a 20 metre by 20 metre grid	Modelled data and GIS	Indices (multiple measures)<br><br>Occupation (lower grade status)<br><br>Education (multiple measures)	Index of Multiple Deprivation<br><br>Individual position from the Whitehall II cohort<br><br>Acorn groups (representing income) in some of the analysis	Relative	**Multivariate with individual and IMD and ACORN factors**<br><br><u>NOx</u><br><br><u>All of London</u><br><br>⊕ Lower grade status<br>n.s. Education, Household income<br><br>⊕ Income (ACORN level)<br><br>⊕ IMD Employment, Education Child,<br>⊖ Education adult

Lavaine 2014	France<br><br>Departments	Departments - 95<br><br>Pop of departments 70,000 to 2 million.<br><br>Total population not provided.	Ecological study, Panel data study 2000 – 2004	NO2, O3, PM10	Monitoring station data	Economic position (Income)<br><br>Indices<br>(Poverty gap: Poverty rate: Standard of living)<br><br>Occupation (unemployed)<br>Education	Census	Absolute and Relative	**Descriptive**<br><br>NO2<br>⊕ Poverty<br><br>Ozone<br>⊕ Poverty<br><br>PM10<br>⊕ Poverty<br><br>**Descriptive (correlations)**<br>NO2<br>⊕ Income. Unemployment. Poverty gap. Standard of living.<br>⊖ Poverty rate<br><br>PM10<br>⊕ Income. Unemployment. Poverty gap. Poverty rate. Standard of living<br><br>O3<br>⊕ Unemployment. Poverty gap. Poverty rate<br>⊖ Income. Standard of living
Lejune et al 2016	Wallonia (Belgium)	Household survey<br>N- 6,018	Study with individual data, cross-sectional study	Air quality index	Municipal level data for air quality tied to individual household level data	Economic position (Income)	Face to face interviews 2012 – 2013	Relative	**Bivariate**<br><br>Air quality<br>⊕Income
Llop S et al 2011	Valencia (Spain)	785 pregnant women	Study with individual data, Cross-sectional study<br><br>(INMA cohort)<br><br>2 questionnaires	N02	93 sampling sites over 4 different sampling periods lasting 7 days each<br><br>GIS data	Education<br><br>Economic position (Social class)<br><br>Other vulnerable groups<br>(Work status at third trimester, Age of mother)<br><br>Ethnicity<br>(Country of origin)	Two questionnaires	Relative and absolute	**Description**<br><br>Home & Home and at work combined<br>⊕ Age of mother (young), Country of origin (Latin America),<br><br>n.s. Social class, Education level, working status 3rd tri<br><br>**Multi-variate linear regression**<br><br>Home & Home and at work combined ⊕ Country of origin (Latin America)<br>⊕Age – youngest more exposed<br>⊕Social class – lowest social class<br><br>n.s. Other Europeans, other social classes,<br><br>**Multiple logistical regressions**<br><br>Home and above NO2 health limit<br><br>⊕Age – youngest more exposed<br><br>⊕ Country of origin (Latin America)<br><br>n.s. Social class<br><br>Time activity and above NO2 health limit<br><br>⊕ Country of origin (Latin America)<br><br>n.s. Age, Social class, not working during third trimester
Milojevic A et al 2017	England	Residential postcodes n – 1,202,578	Ecological study, cross-sectional	Ozone<br><br>Particulate matter (PM10, PM2.5, PM2.5-	5km modelled grid	Indices<br>(Deprivation – income and employment)<br><br>Place of residence	Index of Multiple Deprivation<br><br>LSOA Rural-urban classification	Absolute and relative	**Description**<br><br>Total PM2.5, PM10, Primary PM2.5, PM2.5-10, Sulphate<br>⊕ DeprivationO3<br>⊖ Deprivation

		52,122,136 population<br><br>Note the total population is being used in this study		PM10, primary, nitrate and sulphate PM2.5)		(Rural-urban)			Nitrate PM2.5<br>= Deprivation (U shaped)<br>**Urban**<br><br>Total PM2.5, Primary PM2.5, Sulphate 2.5, PM2.5-10, PM10<br>⊕ Deprivation<br>Nitrate PM2.5<br>= Deprivation<br><br>Ozone<br>⊖ Deprivation<br><br>**Rural**<br><br>Total PM2.5 , Sulphate 2.5, PM2.5 – 10, PM10,<br>⊕ Deprivation<br><br>Nitrate PM2.5, Primary PM2.5<br>= Deprivation<br>Ozone<br>⊖ Deprivation
Mitchell et al 2015	Great Britain	42,000 Lower super output areas and data zones<br><br>Population in 2001 – 57.1 million<br><br>Population in 2011 – 61.3 million	Ecological study (entire country), Panel study (2001 and 2011)	NO2, PM10<br>For 2001 and 2011	1km modelled grid	Indices<br>(Deprivation)	Townsend Index	Relative and absolute	**Description**<br><br>NO2 2001 and 2011<br>⊕ Deprivation<br>PM10 2001 and 2011<br>⊕ Deprivation<br>Reduction in NO2 2001 - 2011⊖ Deprivation<br><br>Increase in PM10 2001 to 2011<br>⊕ Deprivation<br>NO2 exceedances 2001 and 2011<br>⊕ Deprivation<br><br>Improvement in NO2 non-compliant in 2001 to compliant in 2011<br>⊖ Deprivation<br><br>Compliant with NO2 in 2001 but not in 2011<br>⊕ Deprivation<br><br>PM10 exceedance 2001 and 2011<br>⊕ Deprivation
Moreno-Jimenez et al 2016	Madrid and Barcelona (UPA), Spain	Census sections (pop 1000 to 2000<br><br>Populations<br><br>Madrid 3,273,049<br><br>Barcelona 1,619,337	Ecological study , cross-sectional	NO2 annual mean 2010 50 metre by 50 metre grid	Monitoring station data<br><br>GIS	Age<br>[Vulnerable age groups – children 0-4 years: elderly (80+)]<br><br>Ethnicity<br>(Immigrants from countries with a lower GDP per capita than the EU African, Asian, Latin American, European).	Census	Relative and absolute	**Description**<br><br>MADRID<br><br>NO2<br>⊕ Age 80+, Asian, Latin American<br><br>⊖ Age (0-4)<br>European, African<br><br>BARCELONA<br><br>NO2<br>⊕ Age 80+, Asian immigrants, African immigrants, Latin American immigrants, European immigrants<br><br>⊖ Age (0-4)<br><br>**Bivariate**<br><br>MADRID

									<u>NO2</u><br>⊕ Age 80+, Asian, Latin Americans<br><br>⊖ Age (0-4)<br>European, African<br><br><u>BARCELONA</u><br><br><u>NO2</u><br>⊖ Age (0-4)<br><br>⊕ Asian, Latin Americans, European, African<br><br>n.s. Age (80+)
Morrision et al 2014	Glasgow, Scotland	279 Intermediate Geography Zones (IGZ) populations ca 4,000 per zone<br><br>Total pop not provided but figures above suggest 1,116,000	Ecological study, cross-sectional	Air quality – NO2, PM10	GIS	Indices (Deprivation)	Scottish Index of Multiple Deprivation 2010<br>(minus the health domain).	Relative	**Bivariate**<br><br><u>PM10</u><br>⊕ Deprivation<br><br><u>NO2</u><br>⊕ Deprivation
Occelli et al 2016	Dunkerque agglomeration, Northern France	102 IRIS CENSUS (pop ca 2000)<br><br>Pop circa 220,000	Ecological study, cross-sectional	18 trace elements collected from the thali of foliose lichen Xanthoria parientina individually and an overall indicator of pollution used<br><br>Manganese, Titanium, Zinc, Aluminium, Antimony, Arsenic, Beryllium, Cadmium, Cobalt, Chrome, Copper, Mercury, Nickel, Lead, Palladium, Platinum, Rhodium, Vanadium,	GIS	Indices (Deprivation)	Census<br><br>Localised disadvantage index	Absolute and relative	**Description**<br><br><u>All elements and integrated pollution index</u><br>⊕ Deprivation<br><br>**Bivariate - Spearman**<br><br><u>For all elements and integrated pollution index</u><br>⊕ Deprivation<br><br>**Bivariate boxplot**<br><u>Integrated pollution index</u><br>⊕ Deprivation
Ouidir et al 2017	France	IRIS CENSUS<br><br>14,921 pregnant women 2011<br>(worth noting that there is some bias in the types of mothers more likely to be excluded)	Study with individual data, Cross-sectional study<br><br>(ELFE French Longitudinal Study of Children – mother child cohort)	PM2.5 , PM10, NO2, on a 1km grid	Regression model	Other vulnerable groups (Maternal age of mother, in a relationship)<br><br>Education<br><br>Indices (Deprivation)<br><br>Place of residence (Urbanisation)	French Longitudinal Study of Children – mother-child cohort<br><br>European Deprivation Index	Absolute and relative	**Descriptive**<br><br><u>PM2.5</u><br>**=** Maternal age, Education, In a relationship<br>⊕ Deprivation<br>⊖ Urbanisation level<br><br><u>PM10</u><br>= Maternal age, Education, In a relationship<br>⊕ Deprivation<br>⊖ Urbanisation level<br><br><u>N02</u><br>= Maternal age, Education, In a relationship<br>⊕ Deprivation<br>⊖ Urbanisation level<br><br>**Descriptive (CART)**<br><br><u>PM2.5</u>

										⊕Deprivation<br>= Education<br><br>PM10<br>⊕ Deprivation<br>= Education<br><br>NO2<br>⊖ Deprivation<br>⊖ Education<br><br>**Bivariate**<br><br>PM2.5<br>⊕ Deprivation<br><br>⊕ Education (U shaped)<br>n.s. Maternal Age<br><br>PM10<br>⊖ Maternal Age (young)<br>⊕ Education (U shaped)<br>⊕ Deprivation<br><br>NO2<br>⊖ Maternal Age (young)<br>⊖ Education (U shaped)<br>⊕ Deprivation<br><br>**Multivariate**<br><br>LARGE CITY CENTRES<br><br>PM2.5, PM10, NO2<br>⊕ Deprivation<br><br>SMALL CITY CENTRES AND SUBURBAN AREAS<br><br>PM2.5, PM10, NO2<br>⊕ Deprivation<br><br>RURAL AREAS<br><br>PM2.5, PM10,<br>⊕ Deprivation<br><br>NO2<br>n.s. Deprivation<br><br>**Multivariate with GAM**<br><br>LARGE CITY CENTRES<br><br>PM2.5, PM10, NO2<br>⊕ Deprivation<br><br>SMALL CITY CENTRES AND SUBURBAN AREAS<br><br>PM2.5, PM10, NO2<br>⊕ Deprivation<br><br>RURAL AREAS<br><br>PM2.5<br>⊖ Deprivation

									PM10 and NO2<br>n.s. Deprivation<br><br>**Multivariate**<br><br>**Multiple linear regression models**<br><br>PM2.5<br>ALL URBANIZATION LEVELS<br><br>n.s .Education, in a relationship,<br>⊕ Deprivation<br><br>NO2<br>LARGE CITY CENTRE<br>n.s. Education<br><br>SMALL CITY CENTRE AND SUBURBAN AREAS<br>⊕ Education (secondary school)<br><br>RURAL AREAS<br>n.s. Education<br><br>ALL URBANIZATION LEVELS<br>⊕ Deprivation, Not in a relationship
Padilla et al 2013	Lille and Lyon, France	IRIS CENSUS<br><br>Lille 1.1 million (506 census blocks)<br><br>Lyon 1.2 million (510 census blocks)	Ecological study, Panel study data 2002 and 2009	NO2 2002-2009	GIS	Indices (Deprivation)	Census<br><br>Deprivation index	Absolute and relative	**Descriptive**<br><br>LILLE<br><br>N02<br>⊕ Deprivation<br><br>LYON<br>NO2<br>⊕ Deprivation (non-linear)<br><br>**Descriptive (Anova)**<br><br>LILLE<br><br>NO2<br>⊕ Deprivation<br><br>LYON<br>NO2<br>⊕ Deprivation (possible nonlinear relationship)
Padilla et al 2016	Nice, France	IRIS CENSUS<br><br>Nice 537,769 (236 census blocks)	Ecological study, cross-sectional	Proximity to major roads with high traffic (proxy)<br><br>Proximity of green space	GIS	Indices (Deprivation)	Census<br><br>Deprivation index	Relative	**Bivariate**<br><br>Proximity to high traffic roads<br>⊕ Deprivation
Padilla et al 2014	Lille, Lyon, Marseille, Paris,<br><br>France	IRIS CENSUS<br><br>Lille 1,193,244 (504 census blocks)<br>Lyon 1,281,971 (511 census blocks)	Ecological study , Panel study data 2002-2009	NO2	GIS	Indices<br>(Deprivation index)<br><br>Occupation<br>(Unemployed, Insecure job, White collar)<br><br>Economic position<br>(Median Income)<br><br>Ethnicity<br>(Immigrants)	Census<br><br>Deprivation index	Absolute	**Description**<br><br>2002 – 2009<br><br>LILLE<br>NO2<br>⊕ Deprivation<br><br>LYON<br>NO2<br>⊕ Deprivation (nonlinear)

		Marseille<br>1,715,096<br>(628 census blocks)<br>Paris<br>10,354,675<br>(census blocks<br>2,749)				Other vulnerable groups<br>(Single Parent, Subsidized housing, Non-home ownership)<br><br>Education<br>(Higher education)<br><br>Data 2002-2005 and 2006-2009			<u>MARSEILLE</u><br>NO_2<br>⊕ Deprivation<br><br><u>PARIS</u><br>NO_2<br>⊖ Deprivation<br><br>**GAM simple regression**<br><br>2002 2005 – NO_2<br><br><u>LYON</u><br>⊕ Non-homeownership in the neighborhood, White collar<br>⊖ Subsidized housing<br>n.s. Immigrants, Single parent, Insecure job, Unemployment, Higher education, Median income<br><br><u>LILLE</u><br>⊕ Immigrants<br><br>n.s. Unemployed, Single Parent, Insecure job, White collar , Higher education, Non-home ownership in the neighborhood, Subsidized housing, Median Income<br><br><u>MARSEILLE</u><br>⊕ White collar , Median income<br>⊖ immigrants,<br><br>n.s. Single parent, Insecure job, Unemployment, Higher education, Subsidized housing, Non-home ownership in the neighbourhood<br><br><u>PARIS</u><br>⊕ Immigrants<br>Non home ownership in the neighbourhood, Median income<br>⊖ Unemployment,<br>n.s. Single parent, Insecure job, White collar, Higher education, Subsidized housing,<br><br>2006 to 2009<br>NO_2<br><br><u>LYON</u><br><br>⊕ Median income<br>⊖ Single parent,<br>n.s Unemployed, Insecure job, White collar, Higher education, Non-home ownership in the neighbourhood , Subsidized housing, Immigrants,<br><br><u>LILLE</u><br>⊕ Immigrants,<br>⊖ Insecure job<br>n.s. Unemployed, Single Parent, White collar, Higher education, Non-home ownership in the neighbourhood , Subsidized housing, Median Income<br><br><u>MARSEILLE</u><br>⊕ White collar, Median income<br>⊖ Single parent, insecure job,<br><br>n.s. Immigrants, unemployment, Higher education, Subsidized housing, Non homeownership in the neighbourhood

									PARIS<br>⊕ Non homeownership in the neighbourhood, Median income<br>⊖ Single parent,<br>n.s. Immigrants, insecure job, unemployment, white collar, higher education,<br><br>**Multivariate GAM**<br><br>NO2 2002 - 2005<br><br><u>LYON</u><br><br>⊕ white collar jobs<br>Non home ownership in the neighbourhood<br>⊖ Subsidized housing<br><br><br><u>LILLE</u><br>⊕ Immigrants ,<br><br>n.s. single parents, insecure job, unemployment, white collar, higher education, subsidized housing, non-homeownership in the neighbourhood<br>, median income<br><br><u>MARSEILLE</u><br><br>⊕ White collar<br>⊖ Immigrants, median income<br><br>n.s. subsidized housing<br><br><u>PARIS</u><br>⊕ Immigrants , non-homeownership in the neighbourhood<br>⊖ Unemployment, median income<br><br><br>NO2 2006 – 2009<br><br><u>LYON</u><br><br>⊖ Single parent, median income<br><br><u>LILLE</u><br>⊕ Immigrants<br>⊖ Insecure job<br><br><u>MARSEILLE</u><br>⊕ white collar jobs<br>⊖ Single parent, insecure job,<br>Median income<br>n.s. unemployment<br><br>PARIS<br>⊕ home ownership<br>⊖ Single parent, median income
Richardson et al 2013	Europe wide<br><br>Between 31 and 17 countries used.	268 NUTS 2 regions (2006) – in some parts of the analysis only 235 or 210 regions were used.	Ecological study, Panel study 2004 - 2008	PM10 daily mean and annual average concentration on a 10km x 10km grid 2004 to 2008	GIS	Economic position (Average primary household income (2004-2008))	Eurostat	Absolute	**Descriptive**<br><br><u>PM10</u><br><br><u>All of Europe 2004 to 2008</u><br><br>⊕ Economic position (Household income)<br><br><u>Western Europe 2004 to 2008</u><br>⊖ Economic position (Household income)

		Mean pop per region 1,848,263. Total pop not provided.							Eastern Europe 2004 to 2007<br>⊕ Economic position (Household income)<br><br>Eastern Europe 2008<br>⊖ Economic position (Household income)<br><br>**Bivariate**<br><br>PM10<br><br>All of Europe 2004 to 2008<br>⊕ Economic position (Household income)<br><br>Western Europe 2004,2007,2008<br>n.s. Economic position (Household income)<br><br>Western Europe 2005, 2006<br>⊖ Economic position (Household income)<br><br>Eastern Europe 2004 to 2008<br>n.s. Economic position (Household income)
Rivas et al 2017	London, UK	Lower Super Output Areas (n not provided)<br><br>Total population circa 8 million	Ecological study, cross-sectional	PM1, PM2.5, pm10, Black carbon (BC), Ultrafine particles (PNCs) during commuting	GIS and Aerosol spectrometer and range of other equipment for personal measurements during commuting	Indices (Deprivation)	2011 Census Special workplace statistics,<br><br>2015 Index of Multiple Deprivation (income deprivation score used)	Absolute	**Descriptive**<br><br>BC<br>n.s. Deprivation<br><br>PNC<br>n.s. Deprivation<br><br>PM1<br>n.s. Deprivation<br><br>PM2.5<br>n.s. Deprivation<br><br>PM10<br>n.s. Deprivation<br><br>**Bivariate**<br><br>BC<br>n.s. Deprivation<br><br>PNC<br>n.s. Deprivation<br><br>PM1<br>⊕ Deprivation<br><br>PM2.5<br>⊕ Deprivation<br><br>PM10<br>⊕ Deprivation
Scharte and Bolte 2013	Bavaria, Germany	Individual 17,218 preschool children	Study with individual data, repeated cross-sectional studies (three 2004 to 2007)	Traffic load at the place of residence:	Self-administered questionnaire	Other vulnerable groups (Single parent)	Three cross sectional surveys 2004 - 2007	Absolute and relative	**Bivariate**<br><br>Traffic Load on residential road<br>⊕ Single mothers cf couple families
Temam et al 2017	16 cities in 7 Western European countries	Cities including census blocks	Ecological study and study with individual data, cross-sectional study	NO2 modelled annual average	Land use regression models Measurements between 2008 and 2011	Occupation (Individual level)<br><br>Education<br><br>Indices (Neighbourhood level	A very large range of various national datasets	Relative	**Multivariate linear regression model with city as level** (Both adjusted for individual factors and mutually adjusted for individual and neighbourhood SEP show the same pattern).<br><br>NO2

	Norwich,<br>Ipswich,<br>Antwerp,<br>Paris,<br>Lyon,<br>Grenoble,<br>Marseille,<br>Geneva,<br>Verona,<br>Pavia,<br>Turin,<br>Oviedo,<br>Galdakao,<br>Barcelona,<br>Albacete,<br>Huelva<br><br>France, Great Britain, Belgium, Switzerland, Italy, Spain	Data on 5,692 individuals	(Three multicenter cohort studies)			Unemployment rate)			<u>Individual Level</u><br>⊖Education (Low)<br>⊖Occupational class (see side note)<br><u>Neighbourhood level</u><br>⊕Unemployment rate<br><br>**Multivariate linear regression model with neighborhood and city level** (Both adjusted for individual factors and Mutually adjusted for individual and neighbourhood SEP show the same pattern)<br><br><u>NO2</u><br><u>Individual level</u><br>n.s. Education, Occupational class<br><u>Neighbourhood level</u><br>⊕Unemployment rate<br><br>**Multilevel linear regression model**<br>**City Level**<br><br><u>NO2</u><br><u>Verona</u><br>⊖Education (Low)<br>Lyon<br>⊖Education (Low)<br><u>Paris</u><br>⊖Occupational class<br>Huelva<br>⊕ Occupational class<br><u>All other cities</u><br>n.s. Education , Occupational class<br><br>**Multilevel logistic regression (education adjusted for neighborhood unemployment)**<br><br><u>NO2 High exposure</u><br><u>All cities</u><br>⊕ Education (Low)<br><br>**Multilevel logistic regression (occupational class adjusted for neighborhood unemployment)**<br><br><u>NO2 High exposure</u><br><u>All cities</u><br>⊕ Occupational class IV<br><br>**Single level linear regression model**<br><br><u>NO2</u><br><u>Lyon, Marseille, Verona, Oviedo, Albacete</u><br>⊖ Education (Low)<br><u>All other cities</u><br>n.s. Education

									<u>Oviedo</u><br>⊖ Occupational class<br><br><u>All other cities</u><br>n.s. Occupational class<br><br>**Multilevel linear regression neighborhood deprivation Adjusted for education**<br><br><u>NO2</u><br><u>Norwich, Paris, Lyon, Grenoble, Marseille</u><br>⊕Deprivation<br><br><u>Oviedo, Albacete</u><br>⊖ Deprivation<br><br><u>Ipswich, Verona, Pavia, Galdakao, Barcelona, Huelva</u><br>n.s. Deprivation<br><br>**Multilevel linear regression neighborhood deprivation Adjusted for occupational class**<br><br><u>NO2</u><br><br><u>Norwich, Paris, Lyon, Grenoble, Marseille</u><br>⊕Deprivation<br><br><u>Oviedo, Albacete</u><br>⊖ Deprivation<br><br><u>Ipswich, Verona, Pavia, Galdakao, Barcelona, Huelva</u><br>n.s. Deprivation
Vrijheid et al 2012	Gipuzkoa, Sabadell and Valencia<br><br>Spain	2081 pregnant women	Study with individual data, cross-sectional study<br><br>(INMA cohort)	NO2	Measured and then land use regression modeling	Economic position (Social class)<br><br>Education<br><br>Ethnicity (Country of birth)	Spanish Classification Nacional de Ocupaciones	Relative	**Multivariate**<br><br><u>NO2</u><br><br><u>Gipuzkoa</u><br>n.s. Social class, Education, Country of birth<br><br><u>Sabadell</u><br>NS Social class, country of birth<br>⊖Education<br><br><u>Valencia</u><br>⊕ Social class,<br>n.s. Country of birth, Education
Xie and Hou 2010	Sheffield, England	339 Lower super output areas (these have ca 1,500 population each)	Ecological study, cross-sectional	Air quality Index for England	1km x 1km grid	Education (Average KS2 score, Average KS3 score, Average KS4 score, , Not staying in education post 16, Not entering higher education, No or low qualifications)<br><br>Economic position (Unemployment benefits, Illness benefits)	Office for National Statistics	Relative	**Bivariate**<br><br><u>Air quality index</u><br>⊖Average KS2 score, Average KS3 score, Average KS4 score, Unemployment benefits, Illness Benefits<br><br>⊕, Not staying in education post 16, Not entering higher education, No or low qualifications,
Cesaroni et al 2010	Rome, Italy	Census blocks with at least 50 in habitants	Ecological study, cross-sectional	Road traffic levels as a proxy, plus the use of buffer zones	GIS	Indices<br><br>Education<br><br>Age	Census<br>Tax register<br><br>Socio-economic index	Absolute and relative	**Description**<br><br><u>All 4 road measures</u><br>⊕ Age (older)

		(average pop 500)<br>4888 census blocks<br><br>Total pop 1,898,898							**Multi- variate (fully adjusted model)**<br>Living less than 50m from a High traffic Road<br>⊕ Age (older)<br>⊖ Indices (non linear)<br>⊖ Education<br><br>Distance from HTR<br>⊖ Age (Oldest)<br>⊖ Indices<br>⊖ Education<br><br>Traffic density<br>⊕ Age (older)<br>⊖ Indices<br>NS Education<br><br>**Multivariate (full adjusted model)**<br><br><u>Residents in railway ring</u><br><br><u>Living at <=50m from HTR</u><br><br>⊕ Age (older), Education (Low)<br><br>n.s. Indices<br><br><u>Residents outside the railway ring</u><br><br><u>Living at <=50m from HTR</u><br><br>⊕ Age (older)<br>⊖ Indices, Education (Low)
Malmqvist et al 2011	Southern Sweden	Individual level 81,110 births	Study with individual data, cross-sectional study	NOx<br>Traffic density	GIS	Ethnicity<br>(Country of origin<br>Maternal age)	Swedish Medical Birth Register	Absolute	**Descriptive**<br><u>NOx</u><br>= Maternal age<br>⊕ Non-Nordic mothers
Fernández-Somoano and Tardon 2014	Asturias, Spain	430 pregnant women	Study with individual data, Cross-sectional study<br><br>(INMA cohort)	NO2<br>Benzene	Passive samplers and modelled data using land use regression.	Economic position<br>(Social class)<br><br>Occupation<br>(Working status)<br><br><br>Other vulnerable groups (Maternal Age)<br><br>Education<br><br>Ethnicity<br>(Nationality of mother)	Questionnaire	Absolute	**Bivariate**<br><u>NO2</u><br>n.s. Age, Country of birth of mother, Education Social class, Working status<br><br><u>Benzene</u><br>n.s. Age, Country of birth of mother, Education, Working status<br>⊕ Social class (non-linear)<br><br>**Multivariate**<br><br><u>NO2</u><br>n.s. Age, Education, Social class, Working status<br><br><u>Benzene</u><br>⊖ Age (older for oldest category only)<br>n.s. Social class, education, Working status
Huss et al 2010	Switzerlan d	4.6 million adults	Study with individual data, cross-sectional study<br><br><br>(Swiss National Cohort)	PM10<br>Proximity to major roads<br>200mx200m grid	Dispersion model on a 200m x 200m grid<br><br>Proximity to major roads network	Gender<br><br>Age<br>(Median age)<br><br>Education<br>(Tertiary education)<br><br>Occupation<br>(Unemployed)<br><br>Ethnicity	Swiss National Cohort	Absolute and relative	**Descriptive**<br><br><u>Distance to main road</u><br><br>⊕ Unemployed, Foreign nationals<br>⊖ Education<br>= Gender , Age<br><br><u>PM10</u><br><br>⊕Unemployed,<br>⊕Foreign nationals

						(Foreign nationals)			⊖Tertiary education<br>= Age, Gender
Morelli et al 2016	Grenoble and Lyon, France	Grenoble pop 670,000<br>Lyon pop 2,120,000	Ecological study, cross-sectional	PM2.5 10mx10m grid	GIS	Indices<br>(Deprivation)	European Deprivation Index	Absolute	**Description**<br><br><u>Grenoble</u><br><u>PM2.5</u><br>⊕ Deprivation (Very small)<br><br><u>Lyon</u><br><u>PM2.5</u><br>⊕ Deprivation Inverted U shaped (again very small)

Our hypothesis is that there is a relationship between poor air quality and low socio-economic position. In the studies examined low socio-economic position has been identified using a range of variables e.g. deprivation, income, poverty rate, income support. To standardize the data extraction and to make it easier to understand we have used a ⊕ where the relationship is in the direction of our hypothesis.
For other indicators of a social dimension that were not directly related to income e.g. education, age, foreign nationals, ethnic characteristics then where those values of these variables indicating social disadvantage are associated with poor air quality we have also used a ⊕.

„=“ = no socioeconomic unequal distribution of *air pollution*
n.s. = not significant
„⊕“ = lower social dimension groups (e.g. more deprived populations) have higher *air pollution levels* or lower distances to road/ higher traffic count / significant association in correlation or multivariate analysis (p-value < 0.05)
„⊖“ = lower social dimension groups (e.g. more deprived populations) have lower *air pollution levels* or greater distances to road/ lower traffic count/ significant association in correlation or multivariate analysis (p-value < 0.05)

Table S4 Table Place of residence

A small amount of studies have carried out a stratification by a characteristic of Place of residence.

Study	Place of residence characteristics	Weight of evidence
Bertin et al	Urban or rural	Urban<br>Deprivation ⊖<br><br>Rural<br>Deprivation ⊖
Fernandez-Somoano et al 2013	Urban <50%<br>Urban >50%	Urban <50%<br>Economic position ⊕<br>Education n.s.<br><br>Urban >50%<br>Economic position n.s.<br>Education n.s.
Llop et al 2011	Home<br>Home and work combined	Home<br>Other vulnerable group ⊕<br>Ethnicity ⊕<br>Economic position n.s.<br>Education n.s.<br><br>Home and work combined<br>Other vulnerable group ⊕<br>Ethnicity ⊕<br>Economic position ⊕<br>Education n.s.
Milojevic et al 2017	Urban or rural	Urban<br>Deprivation ⊕<br><br>Rural<br>Deprivation ⊕<br><br>Note the pollutant ozone shows the opposite pattern in both rural and urban areas with deprivation ⊖
Ouidir et al 2017	Large city centres<br>Small city centres and suburban areas<br>Rural areas	Large city centres<br>Indices (Deprivation) ⊕<br>Education n.s.<br><br>Small city centres and suburban areas<br>Indices (Deprivation) ⊕<br>Education ⊕<br><br>Rural areas<br>Indices (Deprivation) – mixed<br>Education n.s.

PRISMA 2009 Checklist

Section/topic	#	Checklist item	Reported on page #
**TITLE**			
Title	1	Identify the report as a systematic review, meta-analysis, or both.	1
**ABSTRACT**			
Structured summary	2	Provide a structured summary including, as applicable: background; objectives; data sources; study eligibility criteria, participants, and interventions; study appraisal and synthesis methods; results; limitations; conclusions and implications of key findings; systematic review registration number.	1
**INTRODUCTION**			
Rationale	3	Describe the rationale for the review in the context of what is already known.	1,2
Objectives	4	Provide an explicit statement of questions being addressed with reference to participants, interventions, comparisons, outcomes, and study design (PICOS).	2
**METHODS**			
Protocol and registration	5	Indicate if a review protocol exists, if and where it can be accessed (e.g., Web address), and, if available, provide registration information including registration number.	2
Eligibility criteria	6	Specify study characteristics (e.g., PICOS, length of follow-up) and report characteristics (e.g., years considered, language, publication status) used as criteria for eligibility, giving rationale.	2,3,4, S1
Information sources	7	Describe all information sources (e.g., databases with dates of coverage, contact with study authors to identify additional studies) in the search and date last searched.	3,4
Search	8	Present full electronic search strategy for at least one database, including any limits used, such that it could be repeated.	3, S2
Study selection	9	State the process for selecting studies (i.e., screening, eligibility, included in systematic review, and, if applicable, included in the meta-analysis).	3,4
Data collection process	10	Describe method of data extraction from reports (e.g., piloted forms, independently, in duplicate) and any processes for obtaining and confirming data from investigators.	3,4
Data items	11	List and define all variables for which data were sought (e.g., PICOS, funding sources) and any assumptions and simplifications made.	3,4,5
Risk of bias in individual studies	12	Describe methods used for assessing risk of bias of individual studies (including specification of whether this was done at the study or outcome level), and how this information is to be used in any data synthesis.	n.a.
Summary measures	13	State the principal summary measures (e.g., risk ratio, difference in means).	6, S1
Synthesis of results	14	Describe the methods of handling data and combining results of studies, if done, including measures of consistency (e.g., I^2) for each meta-analysis.	6, S1

PRISMA 2009 Checklist

Section/topic	#	Checklist item	Reported on page #
Risk of bias across studies	15	Specify any assessment of risk of bias that may affect the cumulative evidence (e.g., publication bias, selective reporting within studies).	n.a.
Additional analyses	16	Describe methods of additional analyses (e.g., sensitivity or subgroup analyses, meta-regression), if done, indicating which were pre-specified.	S4
**RESULTS**			
Study selection	17	Give numbers of studies screened, assessed for eligibility, and included in the review, with reasons for exclusions at each stage, ideally with a flow diagram.	4,5,6
Study characteristics	18	For each study, present characteristics for which data were extracted (e.g., study size, PICOS, follow-up period) and provide the citations.	3,4 S3
Risk of bias within studies	19	Present data on risk of bias of each study and, if available, any outcome level assessment (see item 12).	n.a.
Results of individual studies	20	For all outcomes considered (benefits or harms), present, for each study: (a) simple summary data for each intervention group (b) effect estimates and confidence intervals, ideally with a forest plot.	n.a.
Synthesis of results	21	Present results of each meta-analysis done, ~~including confidence intervals and measures of consistency~~.	10,12,13
Risk of bias across studies	22	Present results of any assessment of risk of bias across studies (see Item 15).	n.a
Additional analysis	23	Give results of additional analyses, if done (e.g., sensitivity or subgroup analyses, meta-regression [see Item 16]).	S4
**DISCUSSION**			
Summary of evidence	24	Summarize the main findings including the strength of evidence for each main outcome; consider their relevance to key groups (e.g., healthcare providers, users, and policy makers).	14,15
Limitations	25	Discuss limitations at study and outcome level (e.g., risk of bias), and at review-level (e.g., incomplete retrieval of identified research, reporting bias).	16,
Conclusions	26	Provide a general interpretation of the results in the context of other evidence, and implications for future research.	14,15, 16,17
**FUNDING**			
Funding	27	Describe sources of funding for the systematic review and other support (e.g., supply of data); role of funders for the systematic review.	18

From: Moher D, Liberati A, Tetzlaff J, Altman DG, The PRISMA Group (2009). Preferred Reporting Items for Systematic Reviews and Meta-Analyses: The PRISMA Statement. PLoS Med 6(7): e1000097. doi:10.1371/journal.pmed1000097

For more information, visit: **www.prisma-statement.org**.
